# Placental Gene Expression in Women with Excessive Gestational Weight Gain

**DOI:** 10.3390/ijms27136041

**Published:** 2026-07-06

**Authors:** Jorge Valencia-Ortega, Renata Saucedo, Erika Magallón-Gayón, Alejandra Contreras-Ramos, Mary Flor Díaz-Velázquez, Debbie López-Sánchez, Clara Ortega-Camarillo, Aldo Ferreira-Hermosillo, Javier Perez-Durán

**Affiliations:** 1 Unidad de Investigación Médica en Enfermedades Endocrinas, Hospital de Especialidades, Centro Médico Nacional Siglo XXI, Instituto Mexicano del Seguro Social, Mexico City 06720, Mexico; jorgevalenciaortega@gmail.com (J.V.-O.); renata.saucedo@imss.gob.mx (R.S.);; 2 Laboratorio de Investigación en Biología Molecular, Hospital Infantil de México “Federico Gómez”, Mexico City 06720, Mexico; acora_ramos@hotmail.com; 3 Tecnológico de Monterrey, Escuela de Ingeniería y Ciencias, Campus Monterrey, Ave. Eugenio Garza Sada 2501 Sur, Monterrey 64700, Mexico; erika.magallon@tec.mx; 4 Hospital de Gineco Obstetricia 3, Centro Médico Nacional La Raza, Instituto Mexicano del Seguro Social, Mexico City 02990, Mexico; mary.diaz@imss.gob.mx; 5 Sección de Estudios de Posgrado, Escuela Superior de Medicina, Instituto Politécnico Nacional, Mexico City 11340, Mexico; debbiearleth21@gmail.com; 6 Unidad de Investigación Médica en Bioquímica, Hospital de Especialidades, Centro Médico Nacional Siglo XXI, Instituto Mexicano del Seguro Social, Mexico City 06720, Mexico; clara.ortega@imss.gob.mx; 7 Departamento de Investigación en Salud Reproductiva y Perinatal, Instituto Nacional de Perinatología “Isidro Espinosa de los Reyes”, Mexico City 11000, Mexico

**Keywords:** gestational weight gain, placenta, transcriptome

## Abstract

Excessive gestational weight gain (EGWG) promotes adverse physiological and molecular changes in the placenta. In the present study, we examined the whole placental transcriptomic profile in women with EGWG to elucidate the molecular basis of EGWG pathogenesis. Whole transcriptome profile was analyzed in placentas from term patients with EGWG compared to controls using RNA-seq. Eight genes were found to be downregulated, and 318 genes were found to be upregulated in the placentas of the EGWG group in comparison with those of the women with adequate gestational weight gain (AGWG). Analysis of differentially expressed genes showed eight biological processes, nine cellular components, and ten molecular functions activated, and ten biological processes, ten cellular components, and ten molecular functions suppressed in the placentas from women with EGWG compared to AGWG. EGWG is characterized by significant alterations in placental gene expression, impacting various biological processes, cellular components, and molecular functions, including activation of chromatin organization and remodeling, nucleoplasm, and histone-modifying activity, and suppression of response to stimulus, organelle, and protein binding.

## 1. Introduction

Gestational weight gain (GWG) is defined as the weight gain experienced by a woman during pregnancy. This weight gain is a natural physiological adaptation that supports the growth and development of the fetus. This process encompasses an increase in both lean body mass, which includes total body water, and fat mass [1]. The Institute of Medicine (IOM), now the National Academy of Medicine (NAM), guidelines are the prevailing classification system for gestational weight gain, categorizing it as inadequate, adequate, or excessive based on pre-pregnancy body mass index (BMI) [2]. Excessive gestational weight gain (EGWG) has been identified as the most prevalent disorder, with prevalence rates ranging from 33.9% to 54.0% across diverse study populations. Inadequate GWG is also frequently observed, with prevalence rates ranging from 19.3% to 34%. Adequate GWG (AGWG) is consistently the least common outcome, with rates typically between 15% and 39.9% [3,4,5,6,7,8].

EGWG has been demonstrated to have a substantial impact on pregnancy outcomes, resulting in elevated risks for both the mother and the offspring. Maternal complications encompass a range of conditions, including gestational hypertension, preeclampsia, gestational diabetes mellitus, cesarean delivery, and postpartum hemorrhage [9,10,11,12,13]. For offspring, EGWG has been associated with delivering large-for-gestational-age infants, macrosomia, and an elevated risk of childhood obesity [9,11,14,15].

Interestingly, EGWG has been associated with increased placental weight, volume, length, breadth, and surface area [16,17,18,19]. It has also been linked to adverse physiological and molecular changes in the placenta, including increased stiffness, altered iron transport, and elevated expression of anti-apoptotic (Bcl-2) and inflammatory (Pim-1) proteins [20,21,22]. Additionally, placental weight has been observed to partially mediate the effects of EGWG on childhood obesity and fetal growth, particularly in term infants [23,24]. However, the identification of the transcriptome profile of the placenta using high-throughput RNA sequencing (RNA-seq) in women with EGWG has not been studied.

In the present study, we examined the complete transcriptomic profile of the placenta in women with EGWG to elucidate the molecular basis of its influence.

## 2. Results

### 2.1. Statistical Analyses

Table 1 summarizes the clinical characteristics of the study groups. The only significant difference between the groups pertained to gestational weight gain.

### 2.2. Differential Gene Expression Profiles of AGWG and EGWG Patients

**Figure 1:**
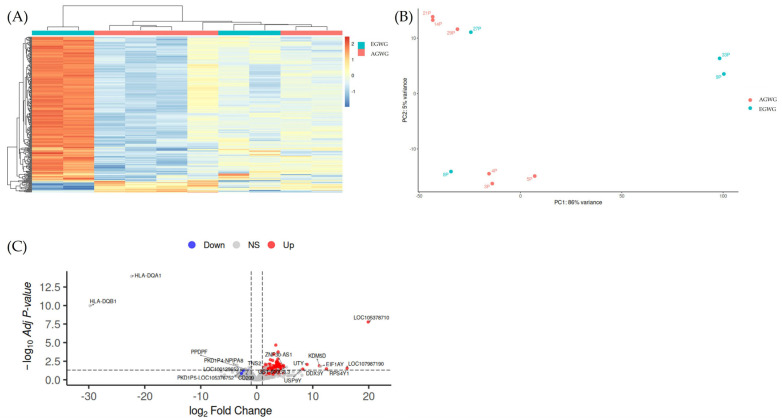
(**A**) **Hierarchical clustering heatmap.** The heatmap shows the gene expression profiles that differ significantly between the AGWG (orange) and EGWG (cyan) groups. Rows correspond to genes and columns to individual samples. Gene expression values are scaled and represented by a color gradient (red for upregulation and blue for downregulation). (**B**) **Principal component analysis (PCA).** PCA was performed on normalized gene expression data to explore the overall variance among samples. Individual samples are represented as distinct points in the graph and color-coded (red for AGWG and cyan for EGWG) to reveal group-wise clustering. (**C**) **Volcano plot.** Blue dots represent genes that passed the adjusted *p*-value significance threshold, while red dots indicate genes meeting both significance and fold change criteria. Vertical dashed lines represent log_2_ fold change cutoffs (±1), and the horizontal dashed line denotes the *p*-value threshold. Genes that demonstrate significant upregulation or downregulation in EGWG in comparison to AGWG are distinctly delineated on either side of the plot. AGWG: adequate gestational weight gain; EGWG: excessive gestational weight gain.

Eight genes were found to be downregulated, and 318 genes were found to be upregulated in the placentas of the EGWG group in comparison with those of the women with AGWG (Figure 1, Supplementary File S1).

### 2.3. Gene Set Enrichment Analysis

The biological processes activated in the placenta of EGWG compared to AGWG were chromatin organization, chromatin remodeling, protein metabolic process, macromolecule biosynthetic process, gene expression, macromolecule metabolic process, biosynthetic process, and metabolic process. The biological processes suppressed were antigen processing and presentation of peptide antigen, T cell activation, lymphocyte activation, regulation of multicellular organismal process, response to stimulus, cell activation, leukocyte activation, positive regulation of cellular process, positive regulation of multicellular organismal process, and positive regulation of biological process (Supplementary File S2).

In cellular components analysis, there was an upregulation of nucleoplasm, nuclear lumen, membraneless organelle, intracellular membraneless organelle, nucleus, membrane-enclosed lumen, organelle lumen, intracellular organelle lumen, and cytosol. Concurrently, there was a downregulation of several components, such as the trans-Golgi network, transport vesicle, transport vesicle membrane, Golgi apparatus subcompartment, side of membrane, bounding membrane of organelle, organelle, organelle membrane, vesicle, and cell periphery (Supplementary File S3).

**Figure 2:**
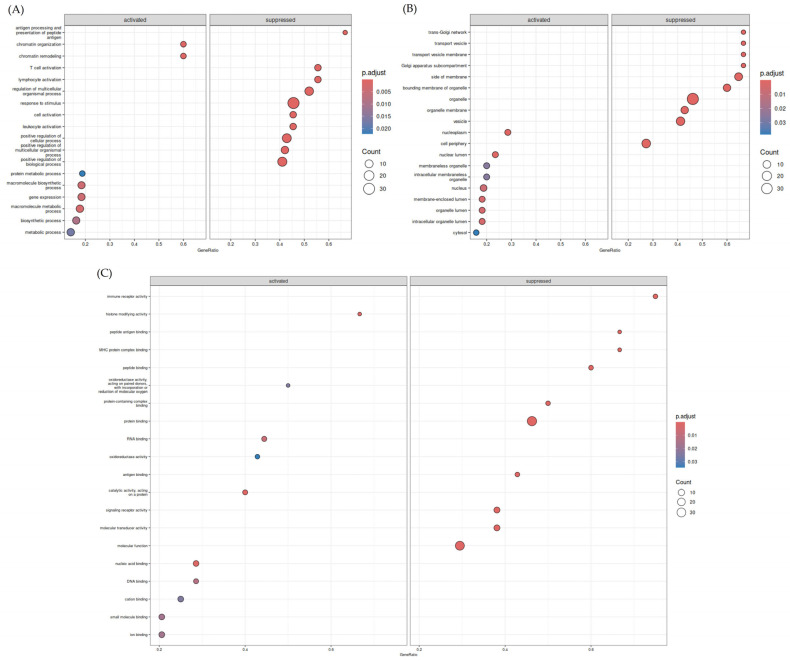
Analysis of differentially expressed genes between EGWG and AGWG groups. Dot plots represent enriched Gene Ontology (GO) terms among differentially expressed genes, categorized into: (**A**) biological process, (**B**) cellular component, and (**C**) molecular function. Terms are bifurcated into activated (upregulated in EGWG) and suppressed (downregulated in EGWG) categories. The size of each data point correlates with the total gene count linked to a specific term, the horizontal axis denotes the gene ratio (proportion of genes involved in the term), and the gradient of color indicates the adjusted *p*-value of enrichment (FDR corrected). AGWG: adequate gestational weight gain; EGWG: excessive gestational weight gain.

Finally, in molecular function, histone-modifying activity, oxidoreductase activity, acting on paired donors with incorporation or reduction in molecular oxygen, RNA binding, oxidoreductase activity, catalytic activity, acting on a protein, nucleic acid binding, DNA binding, cation binding, small-molecule binding, and ion binding were activated. Meanwhile, immune receptor activity, peptide antigen binding, MHC protein complex binding, peptide binding, protein-containing complex binding, protein binding, antigen binding, signaling receptor activity, molecular transducer activity, and molecular function were suppressed in EGWG compared to AGWG (Supplementary File S4) (Figure 2).

## 3. Discussion

To our knowledge, this is the first study to evaluate the placental transcriptome of women with EGWG compared to AGWG. Our RNA-seq analysis revealed significant alterations in placental gene expression in women with EGWG, impacting various biological processes, cellular components, and molecular functions. In EGWG placentas, there was an upregulation of biological processes related to chromatin organization, chromatin remodeling, protein metabolic processes, macromolecule biosynthetic processes, gene expression, and general metabolic processes. Conversely, processes associated with immune responses, such as antigen processing and presentation, T cell activation, lymphocyte activation, and leukocyte activation, were suppressed. Furthermore, EGWG placentas exhibited an upregulation in components like the nucleoplasm, nuclear lumen, membraneless organelle, and nucleus, while showing a downregulation in structures related to transport vesicles, the trans-Golgi network, and the cell periphery. In addition, activated molecular functions in EGWG placentas included histone-modifying activity, oxidoreductase activity, RNA binding, nucleic acid binding, and various cation and small-molecule binding activities. Suppressed functions were predominantly linked to immune receptor activity, peptide antigen binding, MHC protein complex binding, and general protein binding.

The observed activation of chromatin organization and gene expression pathways, alongside increased protein and macromolecule biosynthetic processes, suggests a shift towards enhanced cellular growth and potentially altered cellular differentiation in the EGWG placenta. It has been reported that maternal pre-pregnancy BMI and gestational weight gain are associated with placental DNA methylation changes [25], pointing to a remodeling of the chromatin.

The concurrent suppression of immune-related biological processes and molecular functions points towards a potential immune dysregulation or an altered immune environment at the maternal–fetal interface in EGWG, which could have implications for placental health and pregnancy outcomes. It has been observed that EGWG is associated with marked placental histopathological alterations, including villous structural disorganization, stromal degeneration, increased fibrin deposition, prominent vascular congestion, and an increased number of syncytial knots. These changes may reflect impaired uteroplacental circulation and reduced placental functional capacity [26], which is consistent with our findings.

The changes in cellular components, particularly the upregulation of nuclear elements and downregulation of transport machinery, further support a metabolic and functional reorientation within the EGWG placenta.

The results of this study suggest that transcriptomic alterations in the placenta may play a role in the adverse obstetric outcomes associated with EGWG; however, it remains unclear whether these alterations contribute to EGWG or are caused by it.

The findings of this study should be interpreted with caution due to the following limitations: the small sample size, the lack of validation for differentially expressed genes and protein expression, and the inclusion of only Mexican patients, which may limit the generalizability of the results to other populations.

## 4. Materials and Methods

### 4.1. Patients and Sample Collection

Five women with EGWG and seven women with AGWG were recruited from Hospital de Ginecología y Obstetricia 3, Centro Médico Nacional La Raza, Instituto Mexicano del Seguro Social. The study was conducted in accordance with the Declaration of Helsinki. All patients provided signed informed consent. The study was approved by the Instituto Mexicano del Seguro Social Review Board (R-2023-785-020). Women with pre-gestational comorbidities such as hypertension, uncontrolled thyroid disorders, diabetes mellitus, autoimmune diseases, kidney and liver diseases, and women with pregnancy-related complications (e.g., gestational diabetes mellitus and preeclampsia) or fetal structural and congenital abnormalities were excluded from the study. Information on maternal age, pre-gestational weight, weight at the end of pregnancy, height, delivery time, and neonatal data, such as sex and birth weight, was obtained from medical records. GWG was determined as the difference between the weight at the end of pregnancy before delivery and the pre-gestational weight, and classified according to the criteria established by the Institute of Medicine [27]. BMI was calculated using the formula weight (kg)/[height (m)]^2^, and classified according to the criteria of the World Health Organization [28].

At the time of cesarean section after delivery of the newborn, four random samples (1 cm^3^) of the central region of the placenta on the maternal side were collected in RNAlater (Thermo Fisher Scientific, Waltham, MA, USA). The samples were immediately snap-frozen in liquid nitrogen for transport and stored at −80 °C until assayed. Indications for cesarean section included breech presentation or prior cesarean section.

### 4.2. RNA Isolation

Total RNA was isolated from placental tissues using the AllPrep^®^ DNA/RNA/Protein Mini Kit (Qiagen Inc., Hilden, Germany) according to the manufacturer’s instructions. Total RNA was purified from each of the four placental areas and pooled equally for sequencing.

### 4.3. RNA Library Sequencing

The RNA integrity was assessed using the QSep 400 instrument (BiOptic, New Taipei City, Taiwan) with an R1 RNA cartridge. The RNA concentration and purity were determined using the Qubit RNA HS Assay Kit (Invitrogen, Carlsbad, CA, USA) and NanoDrop 1000 spectrophotometer (Thermo Fisher Scientific, Wilmington, DE, USA), respectively. Transcriptome libraries were prepared using the TruSeq Stranded Total RNA Library Prep with Ribo-Zero Gold (Illumina, San Diego, CA, USA), with fragmentation times adjusted according to RIN. Library quantification was performed using the Qubit dsDNA HS Assay Kit (Invitrogen, Carlsbad, CA, USA), and fragment size was determined on the QSep 400 (BiOptic, New Taipei City, Taiwan). The sequencing was performed on the NovaSeq 6000 (Illumina, San Diego, CA, USA) using a 100 bp paired-end configuration.

### 4.4. Read Quality Assessment

The raw reads were subjected to a quality assessment utilizing the FastQC 0.12.1 software. All raw sequences satisfied the quality control standards.

### 4.5. Alignment and Gene-Level Quantification of Expression

The reads from all libraries were aligned to the reference human genome (GRCh38) using STAR 2.7.11 [29]. Following the mapping stage, gene-level quantification was conducted using the HTSeq 2.0.5 package [30]. The generated count table was used in differential expression analysis.

### 4.6. Differential Expression and Gene Set Enrichment Analysis

After preprocessing the placenta libraries (e.g., normalization and filtering), principal component analysis (PCA) revealed that one control and one problem sample exhibited anomalous behavior. These samples were removed from further analysis. To perform differential gene expression analysis between the EGWG and AGWG groups, the DESeq2 package, which is integrated into the online package iDEP 2.0, was utilized. The identification of differentially expressed genes (DEGs) was conducted through the implementation of a false discovery rate (FDR) threshold of <0.05 and a fold change of >1. Subsequent to this, the DEGs underwent gene set enrichment analysis, which was based on Gene Ontology (GO) databases using all namespaces: molecular function (MF), biological process (BP), and cellular component (CC). This analysis was performed using the gseGO function in the clusterProfiler 4.12.6 package.

### 4.7. Statistical Analysis

Data distribution was evaluated through the implementation of the Shapiro–Wilk test. The differences in characteristics between groups with normal distribution were analyzed using the independent t-test and the chi-squared test. The results are presented as mean ± standard deviation. For this study, statistical significance was attributed to differences with *p*-values below 0.05. The data analysis was conducted using IBM SPSS Statistics 23.0 (IBM SPSS Inc., Chicago, IL, USA).

## 5. Conclusions

EGWG is characterized by significant alterations in placental gene expression, impacting various biological processes, cellular components, and molecular functions, including activation of chromatin organization and remodeling, nucleoplasm, and histone-modifying activity, and suppression of response to stimulus, organelle, and protein binding.

## Figures and Tables

**Table 1:** Clinical characteristics of the study groups.

Characteristic	AGWG (*n* = 6)	EGWG (*n* = 4)	*p*
**Age (years)**	26.8 ± 5.8	32.0 ± 3.9	0.10
**Pre-pregnancy BMI (kg/m^2^)**	21.7 ± 2.4	22.1 ± 2.6	0.55
**Gestational weight gain (kg)**	11.8 ± 2.0	16.8 ± 1.6	0.009
**BMI at delivery (kg/m^2^)**	26.1 ± 2.2	28.5 ± 1.9	0.07
**Gestational age at delivery (weeks)**	37.8 ± 0.9	37.3 ± 0.5	0.43
**Birthweight of newborn (g)**	3055 ± 341	3320 ± 294	0.06
**Fetal Sex (*n*)**			0.43
**Female**	3	3	
**Male**	3	1	

BMI: body mass index; AGWG: adequate gestational weight gain; EGWG: excessive gestational weight gain.

## Data Availability

The original data presented in the study are available in the article and Supplementary Materials. FASTQ files can be provided by contacting the corresponding author.

## Supplementary Materials

The following supporting information can be downloaded at: https://www.mdpi.com/article/10.3390/ijms27136041/s1.
